# Manipulation of Host Microtubule Networks by Viral Microtubule-Associated Proteins

**DOI:** 10.3390/v14050979

**Published:** 2022-05-06

**Authors:** Dahee Seo, Don B. Gammon

**Affiliations:** Department of Microbiology, University of Texas Southwestern Medical Center, Dallas, TX 75390, USA; dahee.seo@utsouthwestern.edu

**Keywords:** virus, cytoskeleton, microtubule, dynein, kinesin, microtubule-associated protein, microtubule-dependent transport, immune evasion, virus–host interactions

## Abstract

Diverse DNA and RNA viruses utilize cytoskeletal networks to efficiently enter, replicate, and exit the host cell, while evading host immune responses. It is well established that the microtubule (MT) network is commonly hijacked by viruses to traffic to sites of replication after entry and to promote egress from the cell. However, mounting evidence suggests that the MT network is also a key regulator of host immune responses to infection. At the same time, viruses have acquired mechanisms to manipulate and/or usurp MT networks to evade these immune responses. Central to most interactions of viruses with the MT network are virally encoded microtubule-associated proteins (MAPs) that bind to MTs directly or indirectly. These MAPs associate with MTs and other viral or cellular MAPs to regulate various aspects of the MT network, including MT dynamics, MT-dependent transport via motor proteins such as kinesins and dyneins, and MT-dependent regulation of innate immune responses. In this review, we examine how viral MAP interactions with the MT network facilitate viral replication and immune evasion.

## 1. Introduction

During virus–host coevolution, viral pathogens are under extraordinary selective pressure to acquire mechanisms to manipulate host machinery to support their replication. The microtubule (MT) network is a web of dynamic cytoskeletal filaments that plays critical roles in eukaryotic cell morphology, division, and intracellular cargo transport [1]. Given the many roles of MTs in key cellular processes, it is not surprising that numerous viruses from diverse families have been found to interact with, and manipulate, MT networks.

Some of the first evidence for virus–MT interactions came soon after the characterization of MTs over 50 years ago [2,3]. For example, in 1963, Dales reported that reovirus replication sites or viral “factories” are found in close proximity to MTs of the mitotic spindle [3]. More recent work has shown that an intact MT network is required for reovirus factory formation, and for its positioning in perinuclear regions of infected cells [4]. In 1975, Luftig et al. showed that adenovirus particles could directly bind to MT filaments in vitro, providing some of the first evidence that viruses may interact with MTs during their life cycle [5]. Subsequently, numerous DNA and RNA viruses have been shown to use MTs for their intracellular transport to replication sites and/or to the cell periphery for exit [6,7]. Furthermore, MT-disrupting drugs such as nocodazole have been shown to suppress infection by a growing number of diverse viruses, highlighting the importance of MTs in virus replication [8,9].

As our understanding of MT regulation and function continues to advance, new roles for the MT network during viral infection have been revealed [10]. For example, recent studies have identified host proteins associated with the MT network as key regulators of the innate immune response to viral infection [11,12]. In turn, new mechanisms of viral manipulation of MT structure and function have additionally been reported, contributing to our understanding of MT regulation and virus–host interactions in general. Central to the interaction between viruses and the MT network is the function of virally encoded proteins. Therefore, in this review, we focus on how virus-encoded proteins directly or indirectly interact with MTs to promote viral replication during different stages of the virus life cycle and how such interactions can also mediate viral immune evasion.

## 2. Functions of the MT Network

MTs are hollow, cylindrical tubes formed by the polymerization of α-and β-tubulin heterodimer subunits [13]. These tubulins can intrinsically self-assemble into filamentous MT structures, but they remain highly dynamic even after their assembly, especially towards the ends that constantly alternate between phases of growth and depolymerization [14]. MT dynamics are tightly regulated by post-translational modifications on tubulin subunits and by MT-associated proteins (MAPs)—factors that may directly or indirectly stabilize, destabilize, and/or reorganize MTs, depending upon the cellular context [14].

MTs are critical for multiple cellular processes. MTs act as a structural scaffold that regulates cell shape and size [15]. In addition, MTs form the mitotic spindle, a collection of MTs that span across the opposite poles of a dividing cell that ensure proper chromosome segregation prior to cell division [16].

Beyond structural and cell division-related roles, the MT network functions as a “roadway” for the intracellular transportation of organelles, vesicles and macromolecules. Two classes of motor proteins called kinesins and dyneins are central to MT-dependent intracellular transport [17]. These motor proteins recognize specific cellular cargo and utilize energy from ATP hydrolysis to “walk” along MTs, providing the basis of MT-dependent transport [17]. One key difference between kinesins and dyneins is that they move in opposite directions along MTs [18]. Kinesins usually carry cargo from the center of the cell towards the periphery in an anterograde manner [18]. In contrast, dyneins tend to move from the cell periphery to the cell center in a retrograde manner [18]. Kinesins and dyneins have both been subdivided into different super families that share a similar mechanism of movement, but have key differences in structure or interaction partners that specify their functions [19,20].

The MT network is also increasingly being recognized to play important roles in regulating innate immunity. The innate immune system is the first line of defense against viral infection. To establish a successful infection, viruses must evade or suppress host innate immunity. To detect viruses, host eukaryotic cells often encode numerous cytosolic or membrane-bound receptors that recognize pathogen-associated molecular patterns (PAMPs), such as cytosolic DNA or viral RNA [21]. PAMP recognition activates transcription factors such as nuclear factor kappa b (NF-κB) and interferon regulatory transcription factor 3 (IRF3) through signaling cascades [21]. These transcription factors, subsequently, stimulate the synthesis and secretion of type I interferons (IFNs) [21]. IFNs are key elements of innate immunity that function in an autocrine and paracrine fashion to establish an antiviral state in the infected cell and uninfected cells nearby after binding to their receptor [21].

One mechanism by which MTs regulate innate immune responses is by mediating the intracellular transport of immunity-related host factors. For instance, MTs are central to the function of Toll-like receptors (TLRs), well-known receptors for PAMPs. It was shown that TLR2 and TLR4 colocalize with MTs in dendritic cells and the disruption of MTs leads to reduced cytokine production upon infection, suggesting these TLRs are transported in an MT-dependent manner during infection [22]. NF-κB, a proinflammatory transcription factor, is also transported to the nucleus in an MT-dependent manner [23]. During viral infection, cytosolic NF-κB is transported to the nucleus by dynein motors, which facilitates downstream proinflammatory and antiviral gene expression [23].

MTs also contribute to innate immunity by regulating the localization of signaling intermediates involved in activating antiviral gene expression. For example, the detection of cytoplasmic viral RNA by the retinoic acid-inducible gene I (RIG-I)-like receptors and melanoma differentiation-associated protein 5 (MDA5) leads to IRF3 activation and subsequent IFN induction [24]. Interestingly, an MT-associated protein, guanidine nucleotide exchange factor H1 (GEF-H1), is a key signaling intermediate in this pathway [11]. Under normal conditions, dynein motors form a complex with GEF-H1 and sequester it on MTs in an inactive form [11]. However, upon RIG-I activation, GEF-H1 is released from MTs into the cytoplasmic space where it can interact with other IFN pathway components to promote signaling [11], suggesting that the GEF-H1-MT association is a key mechanism regulating host antiviral responses.

Lastly, MTs and cellular MAPs contribute to host immune responses through their roles in autophagy. Host cells often activate autophagy during viral infection to target viral particles and proteins for degradation [25]. A key regulator of autophagy, light chain 3 (LC3), was originally discovered as a cellular MAP that directly binds to MTs [26]. During autophagy activation, a MT-associated form of LC3, LC3-I, is lipidated to form LC3-II, and is recruited to the surface of autophagosomes to target them for lysosomal degradation [27]. These LC3-II-marked autophagosomes are transported on MT tracts via dyneins, while lysosomes are transported along MTs by kinesin-1. These bidirectional movements of autophagosomes and lysosomes enable their fusion into autolysosomes, leading to the degradation of autophagosomal contents [28]. As illustrated by the examples above, MTs and MAPs clearly play critical roles in host innate immune responses, but it is unclear if viral MAPs can perform a similar regulation (or deregulation) of these host immune responses.

## 3. Cellular and Viral MAPs

To perform the diverse MT-related functions mentioned above, cells encode MAPs that interact with the MT network for specific roles [29]. MAPs are minimally defined as proteins that interact with MTs, but can be further classified into five different classes depending on their localization and mechanism of action [29,30].

Class I MAPs, such as kinesins and dyneins, are motile MAPs that directly bind to, and move along, the MT filament to facilitate intracellular cargo transport [30]. Class II MAPs include proteins that break or depolymerize MTs, such as the katanin family of MT-severing proteins [30,31]. Class III MAPs include factors such as the XMAP215 family of proteins that act as MT nucleators [30,32]. Class IV MAPs include proteins specifically bound at either end of MTs, such as the capping protein EB1, which binds to highly unstable MT ends to regulate the association and dissociation of tubulin dimers, thereby altering MT dynamics [30,33]. Finally, class V MAPs are nonmotile proteins that, like class I and II MAPs, can bind along the body or lattice of MT filaments, but that typically lack intrinsic enzymatic activity. These MAPs are also known as structural MAPs and they include proteins such as MAP1, MAP2, MAP4, and Tau, and were the first class of MAPs to be discovered through in vitro MT polymerization reactions with brain homogenate in the 1970s [34,35]. Among these class V MAPs, the MAP1 and MAP2/Tau protein families are predominantly restricted to neurons, whereas MAP4 family members are present in many other cell types [36,37,38]. Given their binding to large portions of the MT lattice, class V MAPs can have a wide range of effects on MT assembly, stability, and structure [36,37,38,39]. In addition, class V MAPs can both positively and negatively regulate MT-dependent transport by either promoting or inhibiting motor protein recruitment to MTs, respectively [40].

Given that cellular MAPs are key regulators of MT dynamics and function, it is perhaps not surprising that many viral pathogens encode their own MAPs to directly manipulate MT networks (Table 1). The first animal virus MAP to be discovered was the herpes simplex virus type 1 (HSV-1)-encoded VP22 protein that colocalizes with MTs and forms MT bundles that are resistant to depolymerization by nocodazole—key hallmarks of known cellular MAPs [41]. However, the specific contribution of VP22–MT interactions to the HSV-1 life cycle is still not clear. HSV-1 is only one of many viruses that induce dramatic MT reorganization, but the mechanism of many of these virus-induced MT changes has not been explored in detail, suggesting there are still many viral MAPs to be discovered [4,42,43].

Among known viral MAPs, some share homology with cellular MAPs, such as Ebola virus VP40, which displays amino acid similarity to MAP2 proteins, and thus these viral MAPs may interact with MTs in a manner similar to the cellular MAPs they mimic. However, other viral MAPs, such as alphacoronavirus spike proteins, have no known homology with cellular MAPs and may interact with MTs by unique mechanisms. Here, using selected examples, we review how viral MAPs may function in virus entry, assembly, egress, and immune evasion. We focus on MAPs encoded by animal viruses, since plant virus MAPs have been discussed elsewhere [68,69].

## 4. Viral MAPs in Virus Entry

After attachment, virus entry into the host cell is the next critical step of the virus life cycle and viruses have evolved different strategies to transport viral genetic material across the cell membrane. Some enveloped viruses and most non-enveloped viruses are internalized by receptor-mediated endocytosis, whereas other enveloped viruses directly release viral capsids into the cytosol by fusing their envelopes with the plasma membrane [70]. Either way, the crowded intracellular environment prevents viral capsids or virus-containing endosomes from reaching their sites of replication by simple diffusion [6].

**Figure 1:**
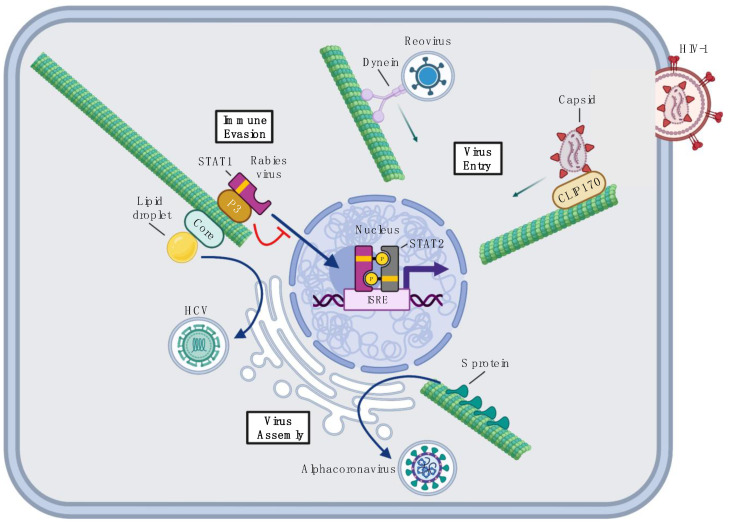
RNA virus and retrovirus interactions with MTs. Examples of how RNA viruses and retroviruses interact with, and manipulate, the MT network. Reovirus uses dynein motors to move towards perinuclear sites to initiate replication. Human immunodeficiency virus type 1 (HIV-1) capsid protein is a cellular MAP mimic that interacts with the cellular MAP CLIP170 to promote its transport to the nucleus via MTs. Hepatitis C virus (HCV) core protein is a MAP that facilitates lipid droplet transport used in virion assembly. Alphacoronavirus spike (S) protein–MT interaction is critical for S protein incorporation onto newly forming virions. Rabies virus evades immune responses by tethering STAT1 to MTs, blocking its association with STAT2 and its nuclear import to activate IFN pathway gene expression. ISRE, interferon-sensitive response element. Figure was created using Biorender.com (accessed on 3 May 2022).

**Figure 2:**
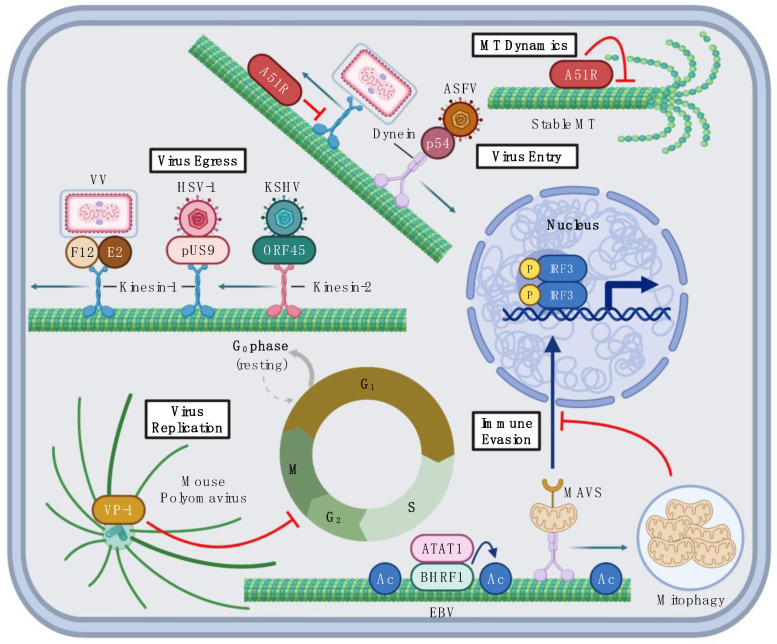
DNA virus interactions with MTs. Examples of how DNA viruses interact with, and manipulate, the MT network. African swine fever virus (ASFV) enters the cell and moves towards perinuclear replication sites by engaging dynein motors through its p54 MAP. Vaccinia virus (VV), herpes simplex virus 1 (HSV-1), and Kaposi sarcoma-associated herpesvirus (KSHV)-encoded MAPs associate with kinesins to facilitate virion egress. VV encodes the viral MAP A51R, that directly binds to, and stabilizes, MTs, as well as blocks kinesin-1 movement on MTs. The mouse polyomavirus VP-1 MAP binds to the mitotic spindle to prevent cell division during infection. Epstein–Barr virus (EBV) evades immune response by hyperacetylating MTs through recruitment of the ATAT1 acetyltransferase using its BHRF1 MAP, which leads to stable MT track formation, and transport of mitochondria to perinuclear sites of mitophagy. This blocks mitochondria-dependent MAVS signaling to IRF3, thereby inhibiting activation of the IFN response. Figure was created using Biorender.com (accessed on 3 May 2022).

To overcome this problem, viruses often hijack dynein motors to use their retrograde movement along MTs to reach perinuclear sites of replication [71,72] (Figure 1 and Figure 2). For example, African swine fever virus (ASFV) binds to dynein motors after initial entry using its envelope protein p54 [47]. ASFV p54 directly binds to dynein light chain 1 in vitro and the depolymerization of MTs by nocodazole or the disruption of dynein function by the expression of dominant-negative inhibitor constructs lead to the inhibition of ASFV infection [47]. These observations suggest that ASFV uses p54 to co-opt dynein motors to promote efficient virion transport to perinuclear regions of the cell where ASFV replication occurs [47] (Figure 2).

Reaching the replication site after initial entry is particularly challenging for neurotropic viruses because initial entry usually occurs at axonal termini, where viral receptors are found, whereas viral replication and assembly sites typically occur in neuronal soma [73]. Therefore, neurotropic viruses must travel along the length of the axon (which can be as long as a meter) to initiate replication, making it difficult for viruses to reach their replication sites by simple diffusion [73]. In order to overcome this challenge, neurotropic viruses, such as herpesviruses, poliovirus, and rabies virus, have acquired mechanisms to utilize dynein motors for the efficient retrograde transport of their virions along the axon, facilitating their transport to their intracellular sites of replication.

More recently, reovirus was also shown to hijack dynein for its retrograde transport in neurons [74]. Although it was previously known that reovirus requires a functional MT network and dynein motors during early stages of infection in non-neuronal cells, their role in neuronal infection was unclear [75]. Aravamudhan et al. showed that reovirus enters the cell by micropinocytosis and is promptly transported in nonacidic vesicles by dynein motors that travel towards the cell soma in primary neuron cultures (Figure 1). After these vesicles reach the soma, they mature and acidify, allowing the virus to disassemble and initiate replication.

Human immunodeficiency virus 1 (HIV-1) is another example of a virus that utilizes MTs during entry. After the initial fusion of HIV-1 envelopes with the plasma membrane, HIV-1 cores travel towards the nucleus to integrate the retroviral genome into the cellular genome. Recent studies showed that HIV-1 cores hijack dynein motors through capsid proteins that directly bind to a dynein adaptor protein Bicaudal D2 (BICD2) [76,77]. BICD2 interacts with the dynactin complex, a multisubunit complex that acts as a linker between dynein and cellular cargo to regulate cargo specificity [76,77]. Therefore, HIV-1 core–BICD2 interactions lead to the indirect tethering of HIV-1 particles to dynein motors for their retrograde transport [76,77]. The depletion of components in the dynein–dynactin–BICD2 complex impairs HIV-1 nuclear import and even increases HIV-1 detection by host innate immune responses, possibly due to the accumulation of HIV-1 particles in the cytoplasm. These results demonstrate that the dynein-dependent transport of HIV-1 is critical for not only HIV-1 entry, but also HIV-1 immune evasion.

Interestingly, HIV-1 utilizes its capsid protein to not only hijack motor proteins on MTs, but also to alter MTs in its favor. In 2013, it was shown that HIV-1 particles rapidly associated with a subset of stabilized MT filaments after its entry that facilitated viral transit to the nucleus [78]. More recently, Da Silva et al. showed that the HIV-1 capsid protein encodes a motif structurally similar to the cellular MAP EB1 [48]. Using this motif, HIV-1 capsids directly bind to the EB1 interactor, CLIP170, which is known to regulate MT dynamics, MT-actin linkages, and the initiation of cargo transport [48,79]. Da Silva et al. suggested that HIV-1 association with CLIP170 enhances infection by promoting virion transport to the nucleus along stabilized MTs [78,80] (Figure 1). This example illustrates how viruses can not only bind to MTs directly, but also encode viral mimics of cellular MAPs to manipulate the MT network for their movement in the host cell after entry.

## 5. Viral MAPs in Virus Replication and Egress

Viral MAPs not only contribute to virus entry, but also facilitate virus replication and the egress of newly formed virions (Figure 1 and Figure 2). One such viral MAP is the hepatitis C virus (HCV) core protein. HCV core is a structural protein that is the main constituent of the HCV nucleocapsid. Mature HCV core consists of two domains: the D1 domain that is involved in RNA binding and the D2 domain that targets the HCV core to lipid droplets (LDs), cellular organelles for lipid storage that are critical for infectious HCV virion production [81]. During infection, the HCV genome is replicated in perinuclear areas by a protein complex bound to the endoplasmic reticulum (ER) and then is packaged into newly formed virions in close proximity to LDs [82]. Interestingly, the D2 domain of HCV core was shown to directly bind to, and stabilize, MTs in vitro, indicating that HCV core protein can function as a MAP [83]. Additionally, HCV core redistributes LDs to perinuclear regions in a MT-dependent manner and this relocalization is crucial for infectious virion production [51] (Figure 1). These findings suggest that HCV core may utilize its interaction with MTs to relocalize LDs to perinuclear areas to facilitate efficient viral genome packaging and virion assembly [51].

Alphacoronavirus spike (S) proteins are another group of viral MAPs that mediate virus assembly. Alphacoronavirus newly formed genomes are packaged into virions at ER-Golgi intermediate compartments close to the nucleus, where the virions acquire envelopes to exit the cell by exocytosis [84]. Rüdiger et al. recently showed that S proteins encoded by several species of alphacoronavirus interact with tubulin by a C-terminal 39 amino acid domain [53] (Figure 1). They also showed that S proteins are usually localized in perinuclear regions during infection, but become dispersed throughout the cytoplasm after nocodazole treatment that disrupts MTs. The disruption of MTs, in turn, leads to reduced S protein incorporation into virions and decreased viral replication. These data suggest that the MT–S protein interactions mediate S protein perinuclear localization, which facilitates its incorporation into new virions, a crucial step in virus assembly [53].

Viruses can manipulate the MT network to not only regulate intracellular transport of virions and viral proteins to replication and assembly sites, but also to alter the cell cycle to promote their replication (Figure 1) [85]. Recently, Horníková et al. showed that the mouse polyomavirus-encoded major capsid protein, VP-1, is a viral MAP that binds directly to, and stabilizes, MTs [86]. They found that VP-1 colocalizes with the mitotic spindle in dividing cells and VP-1 expression in noninfected cells leads to cell cycle arrest at the G2/M phase [58] (Figure 2). These results suggest that VP–1–MT interaction blocks cell division to enable the completion of virus assembly [58,86].

After viral replication, new virions need to be shuttled to the cell surface for successful egress. Therefore, many viruses exploit the anterograde movement of kinesins to move to the cell surface (Figure 2). After initial infection, HSV-1 establishes latency in neurons [87]. However, during reactivation, HSV-1 undergoes its full life cycle and starts producing new virions that require transportation along the axon to neuronal tips for cell exit [88]. To move along the axon, HSV-1 encodes the viral envelope protein pUS9, which binds directly to kinesin-1 motors and facilitates HSV-1 anterograde transport to the axonal periphery, where virions are released [44,88]. Likewise, Kaposi’s sarcoma-associated herpesvirus (KSHV) also moves on MTs to reach the cell surface. Open Reading Frame 45 (ORF45) is a KSHV viral matrix protein that was shown to coimmunoprecipitate with the KIF3A protein, a subunit of kinesin-2 motors [45]. Interestingly, expressing nonfunctional KIF3A or silencing KIF3A by short hairpin RNA significantly decreases the number of extracellular KSHV, but not HSV-1, virions [45]. This suggests that KSHV and HSV-1 use independent mechanisms for their egress, despite both being members of the *Herpesviridae* family (Figure 2).

*Poxviridae* is another family of viruses that heavily utilize MTs for their replication and virion transport. For example, vaccinia virus (VV) F12 and E2 proteins bind to kinesin-1 and tether a subset of VV virions to kinesin-1 for their transport towards the cell surface for exit [63,64,65] (Figure 2). Interestingly, VV infection also induces changes in MT network structure and dynamics [66,89]. For example, Ploubidou et al. reported that VV infection stabilizes a subset of perinuclear MTs and protects them from depolymerization by nocodazole, a phenotype typically observed with cellular MAPs [66]. While this study identified VV-encoded A10L and L4R as viral MAPs that mediate the interaction between the VV core and MTs [66], they concluded that the gross changes in MT dynamics could not be exclusively due to actions of these proteins, since A10L and L4R only associated with acetylated MTs, a small subset of the cellular MT network [66]. Our work, subsequently, showed that the formation of nocodazole-resistant MTs during VV infection to be solely dependent on the expression of the VV A51R protein [90]. A51R proteins are well-conserved among vertebrate poxviruses and the overexpression of A51R proteins encoded by divergent poxviruses is sufficient to promote MT stabilization and bundling [90]. More recently, we have shown that A51R proteins interact directly with MTs through a conserved motif with similarity to cellular Tau proteins, indicating that A51R proteins are a new family of viral MAPs that mimic cellular MAPs [67]. Like Tau, we found A51R proteins to modulate kinesin-dependent transport along MTs in vitro and in mammalian cells, suggesting that viral MAPs may also be key regulators of intracellular cargo transport [67] (Figure 2).

Interestingly, while VV promotes the hyperstabilization of perinuclear MTs, MTs in the cell periphery become more dynamic during infection. This was shown to be due to the function of the VV F11L protein that interacts with RhoA, a Rho GTPase that functions in a signaling network that controls MT dynamics and organization [91]. F11L prevents RhoA from interacting with its downstream effectors that stabilize MTs. Thus, although F11L is not a MAP, it can indirectly affect MT dynamics in the cell periphery in a manner that may facilitate virion release at the cell surface [92].

## 6. Viral MAPs in Immune Evasion

Given the importance of MTs in the host immune response, viruses have acquired strategies to alter or usurp MT networks to facilitate immune evasion (Figure 1 and Figure 2). For example, the rabies virus P3 protein can simultaneously associate with MTs and STAT1, effectively tethering STAT1 to the MT lattice [61]. STAT1 is a key transcription factor involved in the IFN response that, upon activation, heterodimerizes with STAT2 to induce antiviral gene expression in the nucleus [93]. P3 can only inhibit IFN signaling in the presence of a functional MT network because treatment of cells overexpressing P3 with MT-depolymerizing drugs abrogates IFN antagonism by P3 [61]. These data suggest that P3 prevents the heterodimerization of STAT1 nuclear import by tethering STAT1 to MTs in the cytoplasm, thereby blocking IFN pathway activation and antiviral gene expression [61] (Figure 1).

Epstein–Barr virus (EBV) was also recently shown to downregulate the IFN response through the manipulation of the MT network. Glon et al. found that the EBV-encoded BHRF1 protein modulates mitochondria organization in an MT-dependent manner [46]. Mitochondria are one of several cellular organelles that move on MT tracts by motor proteins and they play a critical role in IFN induction through the activity of the mitochondrial antiviral signaling (MAVS) protein, an essential signaling intermediate of the IFN signaling cascade that localizes to the outer mitochondrial membrane [94]. BHRF-1 colocalizes with MTs, particularly acetylated tubulin, and induces the hyperacetylation of MTs by interacting with the host acetyltransferase, ATAT1 [94]. This hyperacetylation promotes the formation of stabilized MTs that facilitate EBV-induced mitochondrial trafficking towards the nucleus using dynein motors [94,95]. The accumulation of mitochondria in perinuclear areas of the infected cell leads to their degradation via mitophagy, resulting in reduced MAVS signaling and the inhibition of the IFN response [94] (Figure 2). Thus, the function of BHRF1 demonstrates how viruses can escape restriction by host immune responses by manipulating MT-dependent transport processes.

## 7. Summary

MTs and MAPs have been the subject of study for decades, ever since the discovery of MTs in the late 1950s [96]. Virus–MT interactions were discovered soon after in the 1960s, and continue to be an active area of research. The examples cited in this review demonstrate some of the diverse strategies viruses employ to manipulate the host MT network to promote their replication. Central to these manipulations are virally encoded MAPs, which are employed by a wide variety of RNA and DNA viruses (Table 1). These MAPs can directly or indirectly associate with MTs and alter MT dynamics and MT-dependent transport to favor viral replication.

Despite these advancements in our understanding of viral MAPs, there are still gaps in our current knowledge that are worth highlighting. For example, recent cryo-EM structures of cellular MAPs bound to MTs have given us tremendous insights into the mechanisms by which MTs are regulated [97]. However, we still do not have structural information for viral MAP-MT interactions. Such information can help to reveal the mechanism by which viral MAPs interact with MT machinery and may elucidate both the similarities and potential differences between how cellular and viral MAPs engage with MTs. Furthermore, although MT colocalization or MT bundling/stabilization phenotypes have been associated with many viral MAPs, the exact reasoning for these functions of viral proteins and the benefit they provide to the virus life cycle remain unclear in most cases and will require further investigation. To our knowledge, our work on A51R is the first to show that viral MAPs can negatively regulate MT-dependent transport. However, it is still unclear if or how other viral MAPs also regulate MT-dependent transport to facilitate viral replication.

For the relatively few viral MAP–MT interactions that have been extensively studied, it is clear that MT networks can influence several aspects of the virus life cycle, such as the entry, replication, and egress. The importance of the MT network to viral replication is underscored by studies showing that the disruption of the MT network with drugs such as nocodazole often reduces viral replication. Moreover, recent studies suggest that the MT network and host proteins associated with MTs can serve important functions in regulating host immune responses to infection. At the same time, mounting evidence suggests that viral MAPs can alter MT functions such as MT-dependent transport or simply use MTs as a platform to tether host immunity factors to counter such immune responses. A deeper understanding of the mechanisms by which viruses manipulate the MT network structure and function may lead to not only the identification of new targets for antiviral therapies, but may also reveal insights into the molecular mechanisms that normally regulate MT networks.

## Figures and Tables

**Table 1:** List of reported viral MAPs.

Virus	Virus Family	Protein	Direct/Indirect Interaction	Reference
Herpes simplex virus type 1	*Herpesviridae*	VP22	Unknown	[41]
Herpes simplex virus type 1	*Herpesviridae*	pUS9	Indirect	[44]
Kaposi’s sarcoma-associated herpesvirus	*Herpesviridae*	ORF45	Indirect	[45]
Epstein–Barr virus	*Herpesviridae*	BHRF1	Unknown	[46]
African swine fever virus	*Asfarviridae*	p54	Indirect	[47]
Human immunodeficiency virus type 1	*Retroviridae*	Capsid	Indirect	[48]
Human immunodeficiency virus type 1	*Retroviridae*	Tat	Direct	[49]
Murine norovirus	*Caliciviridae*	NS3	Unknown	[50]
Hepatitis C virus	*Flaviviridae*	Core	Direct	[51]
Hepatitis C virus	*Flaviviridae*	NS3	Unknown	[52]
Hepatitis C virus	*Flaviviridae*	NS5A	Unknown	[52]
Alphacoronavirus	*Coronaviridae*	Spike (S)	Direct	[53]
Murine coronavirus	*Coronaviridae*	Nucleocapsid	Direct	[54,55]
Rotavirus	*Reoviridae*	NSP2	Unknown	[56]
Rotavirus	*Reoviridae*	NSP5	Unknown	[56]
Rotavirus	*Reoviridae*	VP4	Unknown	[57]
Mouse polyomavirus	*Polyomaviridae*	VP-1	Direct	[58]
Vesicular stomatitis virus	*Rhabdoviridae*	Matrix	Direct	[59]
Chandipura virus	*Rhabdoviridae*	Matrix	Unknown	[60]
Rabies virus	*Rhabdoviridae*	P3	Unknown	[61]
Ebola virus	*Filoviridae*	VP40	Direct	[62]
Vaccinia virus	*Poxviridae*	F12L	Indirect	[63,64,65]
Vaccinia virus	*Poxviridae*	E2	Indirect	[63]
Vaccinia virus	*Poxviridae*	A10L	Direct	[66]
Vaccinia virus	*Poxviridae*	L4R	Direct	[66]
Vaccinia virus	*Poxviridae*	A51R	Direct	[67]
